# Development and Validation of a Real-Time PCR Based Assay to Detect Adulteration with Corn in Commercial Turmeric Powder Products

**DOI:** 10.3390/foods9070882

**Published:** 2020-07-05

**Authors:** Su Hong Oh, Cheol Seong Jang

**Affiliations:** Plant Genomics Laboratory, Department of Bioresource Sciences, Kangwon National University, Chuncheon 24341, Korea; ohsuhong@kangwon.ac.kr

**Keywords:** anti food fraud, *Curcuma longa*, DNA markers, species identification, SYBR-GREEN real-time PCR, *Zea mays*

## Abstract

Turmeric, or *Curcuma longa*, is commonly consumed in the South East Asian countries as a medical product and as food due to its therapeutic properties. However, with increasing demand for turmeric powder, adulterated turmeric powders mixed with other cheap starch powders, such as from corn or cassava, are being distributed by food suppliers for economic benefit. Here, we developed molecular markers using quantitative real-time PCR to identify adulteration in commercial turmeric powder products. Chloroplast genes, such as *matK, atpF*, and *ycf2*, were used to design species-specific primers for *C. longa* and *Zea mays*. Of the six primer pairs designed and tested, the correlation coefficients (R^2^) were higher than 0.99 and slopes were −3.136 to −3.498. The efficiency of the primers was between 93.14 and 108.4%. The specificity of the primers was confirmed with ten other species, which could be intentionally added to *C. longa* powders or used as ingredients in complex turmeric foods. In total, 20 blind samples and 10 commercial *C. longa* food products were tested with the designed primer sets to demonstrate the effectiveness of this approach to detect the addition of *Z. mays* products in turmeric powders. Taken together, the real-time PCR assay developed here has the potential to contribute to food safety and the protection of consumer’s rights.

## 1. Introduction

Turmeric (*Curcuma longa*) belongs to the ginger family, Zingiberaceae, and is native to Southern Asia and India. Turmeric rhizomes, which have brown skin and a unique flavor, are commonly used as a coloring and flavoring agent in Asian cuisines. Due to its fragrant aroma and slightly bitter taste, turmeric is a common culinary spice in Indian cuisines, especially curry. Additionally, beyond food products, turmeric is commonly consumed as a medical product in South East Asian countries due to its therapeutic properties [1]. The market size of curcumin was valued at USD 58.4 million in 2019 and is expected to experience a CAGR (compound annual growth rate) of 12.7% from 2020 to 2027 [2]. Globally, the demand for turmeric has grown due to its therapeutic functions and low toxicity. Curcumin, (1,7-bis(4-hydroxy-3-methoxyphenyl)-1,6-heptadiene-3,5-dione), also known as diferuloylmethane, is the main natural polyphenol found in rhizomes of *C. longa* (turmeric) and in other *Curcuma* spp. [3]. It has been shown to target multiple signaling molecules while also demonstrating activity at the cellular level, which has helped support its multiple health benefits [4]. It has beneficial effects in inflammatory conditions [3], metabolic syndrome [5], and pain [6], as well as helps in the management of inflammatory and degenerative eye conditions [7]. While there appear to be countless therapeutic benefits of curcumin supplementation, most of them may be due to its antioxidant and anti-inflammatory effects [3].

Reports on the medicinal value of turmeric in treating a variety of ailments have further increased the global demand for turmeric [8]. In the United States, the largest market for turmeric supplements, turmeric was the top-selling herbal supplement, with sales exceeding US $47.6 million in 2016 [8,9]. In addition, turmeric-based dietary supplements, which also include standardized extracts with high concentrations of curcumin, have seen a steady increase in popularity in the United States and elsewhere [10,11]. However, with the increasing demand for turmeric powder, adulterated turmeric powders mixed with other cheap starch powders, such as from corn or cassava, have been distributed by food supplies for economic benefit [12]. According to the United States Grocery Manufacturers Association, food fraud costs $10–15 billion annually in the global food industry and affects approximately 10% of all commercial foods sold [13]. 

To detect fraudulent ingredients in complicated mixed foods, various technologies, such as sensory-, physicochemical-, chromatographic-, spectroscopic-, and DNA-based assays have been developed. DNA is generally believed to be stable enough to withstand various chemical treatments and high temperatures, and small quantities of DNA can be detected with specific primers using PCR-based methods [14]. DNA-based methods, such as quantitative real-time PCR (real-time PCR), multiplex PCR, and PCR-RFLP have been successfully applied to detect food fraud and adulteration due to their economical and time-saving advantages over other approaches [15,16].

Specifically, real-time PCR (real-time PCR) assay presents with high specificity and sensitivity, capable of detecting very small amounts of target DNA in complex foods. General types of real-time PCR approaches, probe-based real-time PCR (TaqMan assay), and DNA intercalating dye-based real-time PCR (SYBR Green I assay) have been employed for the detection and identification of DNA [17]. Probe-based real-time PCR detects the target sequence with specificity using probes designed to be complementary to a target sequence [18]; however, this approach requires many SNPs or indels to differentiate species, and it is difficult to design probes and optimize real-time PCR conditions [19,20]. Alternatively, SYBR Green I, an intercalating dye that binds to double-stranded DNA in a sequence independent manner, can provide a more flexible, convenient, and inexpensive method over probe-based methods [19].

It is generally believed that the nuclear genome of a cell has a single copy of a particular gene along with a few sequences in low copy numbers; hence, it is difficult to obtain high uniformity in PCR amplification. Especially, DNA extracted from processed commercial foods is of low quality, possibly because of degradation caused by the processes of drying, heating, fermentation, and addition of ingredients. Therefore, markers designed on the extracted nuclear DNA from processed foods exhibit a low ability to discriminate between species because of the low quality of nuclear DNA that has either a single gene or low-copies of genes [21,22]. The chloroplast genome size varies among species, ranging from 107 to 208 kb and consisting of a single circular molecule of DNA that is generally present in hundreds of copies per cell [23]. Chloroplasts are composed of two layers of membranes that enable chloroplasts to persist through decomposition during food processing [24]. The chloroplast genome is generally believed to contain 120–130 genes [22]. Some genes, such as *matK*, *ndhF*, *ycf*2, and *ccsA*, exhibit higher frequencies of single-nucleotide polymorphisms (SNPs) and insertion/deletions (indels) than other chloroplast genes [23]. A variety of chloroplast markers, including *atpF*-*atpH* spacer, *matK* gene, *rbcL* gene, *rpoB* gene, *rpoC1* gene, *psbK-psbl* spacer, and *trnH-psbA* spacer, have been employed for species identification [25,26].

As described above, cheap corn powder with a similar color to turmeric has been wildly used in adulterated turmeric powders by food suppliers for illegal economic benefit. In this study, we developed SYBR Green-based quantitative real-time PCR assay to identify adulteration in commercial turmeric powder products using turmeric and corn species-specific primer sets. The real-time PCR methodology was optimized for both species-specific primers to correctly identify target species in complex powder products. Subsequently, the designed primers were applied to commercial turmeric products.

## 2. Materials and Methods

### 2.1. Plant and Food Sample Preparation

Turmeric (*Curcuma longa*) rhizomes and corn (*Zea mays*) seeds were kindly provided by Gangwondo Agriculture Research and Extension Services (Chuncheon, Korea). Both plants were grown in a stable temperature greenhouse for four weeks with horticulture soil. Samples for DNA isolation were extracted from the leaves of each plant. All *C. longa* commercial products used for the analysis of food complexes were purchased from local markets and stored at room temperature. 

#### 2.1.1. Reference Binary Mixtures

To generate a quantitative reference binary mixture model, binary mixtures containing different amounts (2 mg, 0.1%; 20 mg, 1%; 200 mg, 10%; and 2 g, 100%) of turmeric rhizome powders were mixed to prepare a final mixture of 2 g with corn powder, wheat flour, or rice flour purchased from a local market. Additionally, different amounts of corn powder were mixed (2 mg, 0.1%; 20 mg, 1%; 200 mg, 10%; and 2 g, 100%) to prepare final mixtures of 2 g with turmeric rhizome powders. Turmeric rhizomes and corn seeds were dried in a 55 °C dry oven for 48 hours and then ground with a mixing machine.

#### 2.1.2. Blind Samples

Blind powder samples (*n* = 20) were provided by the National Institute of Food and Drug Safety Evaluation of the Ministry of Food and Drug Safety (Cheongju, Korea). The blind samples consisted of different percentages of corn and turmeric rhizome powders. The corn powders were added to turmeric rhizome powders at concentrations of 0–10% *w*/*w*, to prepare final mixtures of 150 mg.

### 2.2. DNA Extraction

For the efficiency of the designed primer sets, genomic DNA used for standard curves was extracted from *C. longa* and *Z. mays* leaves using the Dneasy Plant Pro Kit (QIAGEN, Hilden, Germany) according to the manufacturer’s protocol. Genomic DNA used to plot standard curves of reference binary mixtures was isolated from the binary mixture samples (2 g each) using a large scale CTAB-based genomic DNA isolation method [27]. Genomic DNA from the commercial turmeric products was extracted using the Dneasy Plant Pro Kit according to the manufacturer’s protocol. To obtain high quality genomic DNA, DNA extracted with the large scale CTAB method was purified using the Wizard DNA Clean-Up system (Promega, Madison, USA). DNA quantity and purity were measured using a SPECTROstar Nano reader (BMG Labtech, Ortenberg, Germany). Purity of the DNA extracts was in the range of 1.7–2.

### 2.3. Sequence Analysis and Primer Design

Sequences of target chloroplast genes such as *matK*, *atpF*, and *ycf2* of two species (*C. longa* for NC_042886.1 and *Z. mays* for NC_001666.2) were downloaded from the National Center for Biotechnology Information (NCBI) and used to design target-specific primers. The nucleotide sequences of the both species were aligned using ClustalW2 (EMBL-EBI, Hinxton, Cambridgeshire, UK) and BioEdit 7.2 (Ibis Biosciences, Carlsbad, CA, USA). Species-specific primer sets were designed based on the variable region between *C. longa* and *Z. mays* using Beacon Designer^TM^ (PRIMER Biosoft, Palo Alto, CA, USA). Species-specific primers were commercially synthesized (Macrogen, Seoul, Korea). 

### 2.4. Quantitative Real-Time PCR

Real-time PCR was performed in a final volume of 20 µL using AccuPower^®^ 2× GreenStar™ real-time PCR Master Mix with SYBR Green (Bioneer, Daejeon, Korea). The real-time PCR reaction mixture consisted of 10 µL 2× GreenStar Master Mix, 0.5 µL 10 pmol each primer, 1 µL of 10 ng/µL genomic DNA, and 0.25 µL ROX Dye. A QuantStudio 3 Real-Time PCR System (Applied Biosystems, Foster City, CA, USA) was used for real-time PCR amplification. The real-time PCR conditions were as follows: pre-denaturation (10 min at 95 °C), followed by 40 cycles of denaturation for 30 s at 95 °C, annealing for 20 s at 55–60 °C (depending on each targeting primer sequence), and extension for 30 s at 72 °C. All real-time PCRs were performed in technical triplicates for three biological replicates.

### 2.5. Cloning of PCR Amplicons and Sequencing

Conventional PCR was carried out using TaKaRa Ex TaqTM DNA polymerase (TaKaRa Bio Company, Kusatsu, Shiga, Japan) mixture with 10 ng DNA and 10 pmol each primer using a C1000 Thermal Cycler (BIO-RAD, California, USA). PCR conditions were as follows: pre-denaturation for 5 min at 95 °C, followed by 35 cycles of annealing and denaturation for 30 s at 95 °C, annealing for 20 s at 55–60 °C (depending on primer sequences), and extension 30 s at 72 °C, and final extension for 5 min at 72 °C. PCR products were amplified using target specific primers (CL_matK, CL_atpF, CL_ycf2, ZM_matK, ZM_atpF, and ZM_ycf2) and cloned using the RBC T&A Cloning Vector (Real Biotech Corporation, Taipei, Taiwan). Plasmid DNA was extracted from recombinant plasmids using the DokDo-Prep Plasmid Mini-Kit (ELPISBIOTECH, DaeJeon, South Korea) and sequenced by a commercial service (Macrogen, Seoul, Korea).

### 2.6. Standard Curve Construction and Data Analysis 

The efficiency of the designed primer sets was evaluated using two approaches. First, species-specific PCR products were cloned into the RBC T&A Cloning Vector (Real Biotech Corporation, Taipei, Taiwan), and recombinant clones were then diluted serially (10^7^, 10^6^, 10^5^, 10^4^, and 10^3^ copies) and used to quantify and confirm the efficiency of equivalent amplification [28,29]. Second, real-time PCR assays were applied to genomic DNA using target and non-target gDNA diluted ten-fold into five series (10 ng to 1 pg).

Each binary mixture with genomic DNAs extracted from the leaves or powder products of each species was diluted to a final concentration of 10 ng/μL. A baseline and a threshold were set for further analysis. The cycle number at the threshold level of log-based fluorescence was defined as the Ct (cycle threshold) number, which was the observed value in the conventional real-time PCR experiments [30]. Correlations between diluted DNAs and cycle threshold (Ct) standard curves were evaluated using a default parameter. The standard curve was calculated as y = −ax + b (a refers to the standard curve slope and b refers to the y-intercept). The efficiency of the reaction (E) was calculated as E = (10^−1/a^), and the percent efficiency was evaluated as (E − 1) × 100% [29,30]. For all analyses, three technical replicates of each biological replicate were performed.

To evaluate amplification efficiency and sensitivity, two criteria were used to define an acceptable real-time PCR assay based on previous reports [28,29]: linear dynamic range and amplification efficiency. The linear dynamic range should ideally extend over four log_10_ concentrations, with the coefficient of determination (R^2^) being greater than 0.98, and the amplification efficiency should be in the range of 110-90%, corresponding to a slope between −3.1 and −3.6 [29].

To validate the specificity and sensitivity of the designed target-specific primers, interlaboratory validation was performed in two independent laboratories. Validation was performed in two laboratories using the same PCR conditions and with either an Applied Biosystems 7500 Fast Real-Time PCR Instrument System (Applied Biosystems, Foster City, CA, USA) or a CFX Connect Real-Time PCR Detection System (Bio-Rad, Hercules, CA, USA).

## 3. Results and Discussion

### 3.1. Design of Species-Specific Primers

To verify authenticity of *C. longa* commercial food products, we designed species-specific primer pairs for *C. longa* and *Z. mays*. Chloroplast genes, such as *matK, atpF,* and *ycf2*, with high frequencies of SNPs and indels between the two species [25] were targeted to design the species-specific primer sets. For designing species-specific primers, chloroplast genes of both species, as well as those of other starch crops (*Oryza sativa* and *Triticum aestivum*), were aligned using a software program with ClustalW2 (EMBL-EBI, Hinxton, Cambridgeshire, UK) and BioEdit 7.2 (Ibis Biosciences, Carlsbad, CA, USA; Supplementary Figures S1 and S2). We identified a variety of SNPs within three chloroplast genes among four species (Supplementary Figure S1). Food processing, such as heating, drying, and mixing, is known to damage and degrade DNA [31]. If the length of PCR amplicons is long, real-time PCR would be decreased in various food products. Therefore, based on species-specific SNPs, target-specific primers were designed to amplify short products ranging from 80 to 194 bp (Table 1).

### 3.2. Amplification Efficiency of the Designed Primer Sets 

**Figure 1:**
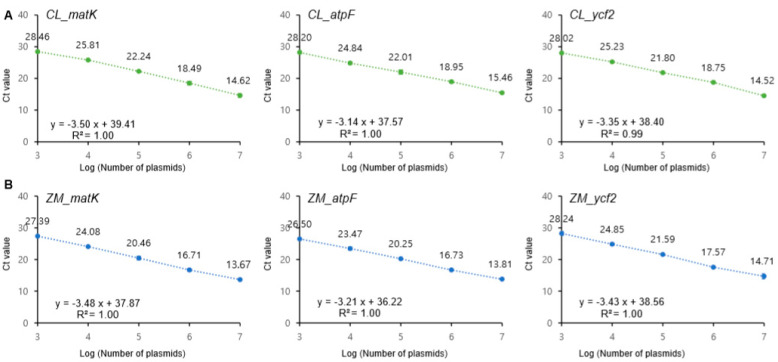
Standard curve of cycle threshold (Ct) values were obtained on the basis of efficiency and correlation of coefficient (*R*^2^) in serial dilution series recombinant plasmids (*C. longa* and *Z. mays*) using species-specific primer sets. The *x*-axis represents log number of plasmids and the *y*-axis represents means of Ct value ± SD. (**A**) *C. longa* targeting primer sets (CL_matK, CL_atpF, and CL_ycf2). Green dots represent serial dilution series of recombinant plasmids (107–103) containing *C. longa* specific target gene (matK, atpF and ycf2) sequences; (**B**) *Z. mays* targeting primer sets (ZM_matK, ZM_atpF, and ZM_ycf2). Blue dots represent serial dilution series of recombinant plasmids (107–103) containing *Z. mays* specific target gene (matK, atpF and ycf2) sequences. The real-time PCRs were carried out in triplicate (*n* = 3).

**Figure 2:**
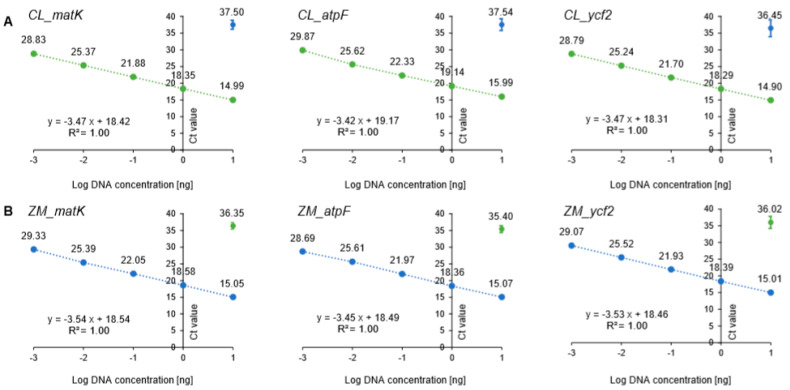
Standard curve of cycle threshold (Ct) values were obtained on the basis of efficiency and correlation of coefficient (*R*^2^) in serial dilution series genomic DNA (*C. longa* and *Z. mays*) using species-specific primer sets. The *x*-axis represents log DNA concentration (ng) and the *y*-axis represents means of Ct value ± SD. (**A**) *C. longa* targeting primer sets (CL_matK, CL_atpF, and CL_ycf2). Green dots represent serial dilution series of genomic DNA in *C. longa* leaves (10ng–1pg) and blue dots represent genomic DNA of *Z. mays* (10ng); (**B**) *Z. mays* targeting primer sets (ZM_matK, ZM_atpF, and ZM_ycf2). Blue dots represent serial dilution series of genomic DNA in *Z. mays* leaves (10ng–1pg) and green dot represents genomic DNA of *C. longa* (10ng). The real-time PCRs were carried out in triplicate (*n* = 3).

Amplification efficiency of the six primer sets (CL_matK, CL_atpF, CL_ycf2, ZM_matK, ZM_atpF, and ZM_ycf2) was evaluated by constructing standard curves using 10-fold serial dilutions (10^7^ to 10^3^) of each recombinant plasmid DNA, and regression analyses were performed (Figure 1, Supplementary Figure S3). The correlation coefficients (R^2^) of the six primer pairs were higher than 0.99, and slopes ranged from −3.14 to −3.50. The efficiency of the primers was between 93.14 and 108.40% (Supplementary Table S1). All values fit the ENGL (European Network of GMO Laboratories) guidelines, with the coefficient of determination (R^2^) being greater 0.98 and the amplification efficiency ranging from 110 to 90%, which corresponds to a slope between −3.1 and −3.6 [29]. Subsequently, we evaluated the efficiency of the primers using the 10-fold serially diluted genomic DNAs (from 10 ng to 1 pg) extracted from plant samples (Figure 2, Supplementary Figure S4). Similarly to the results of recombinant plasmid DNAs, standard curves in the gDNA samples also ranged from −3.42 to −3.54, exhibited *R*^2^> 0.99, and efficiency values of 91.78–95.92%, which also conformed to the ENGL guidelines (Supplementary Table S1) [29].

In addition, to evaluate the adaptability of the primes across machines, amplification efficiency was evaluated by two independent laboratories. As a result, the primer sets were found to meet the ENGL criteria (R^2^ > 0.98 and efficiency ranges of 91.78–108.40; Supplementary Table S2). Based on the evaluation of amplification efficiency of the designed primer sets through three approaches, with recombinant plasmids, genomic DNA, and interlaboratory evaluation, the designed primer sets could be suitable to detect the target species.

### 3.3. Sensitivity and Specificity of the Assay

**Figure 3:**
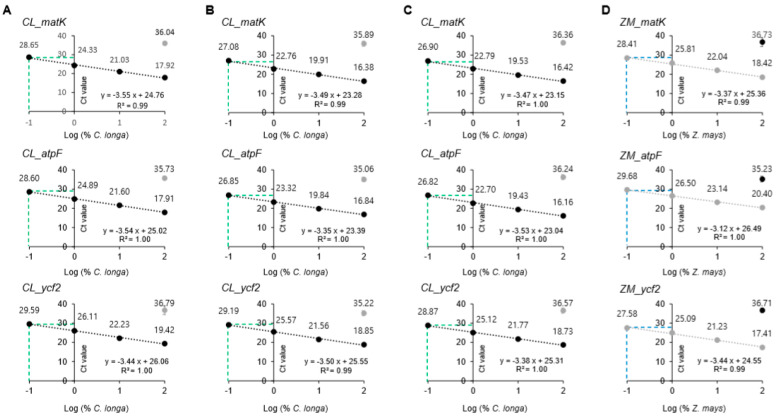
Standard curve of cycle threshold (Ct) values obtained on the basis of efficiency and correlation of coefficient (*R*^2^) by reference binary mixtures. The *x*-axis represents log percentage of the target species (%) and the *y*-axis represents means of Ct value ± SD. plotted against the logarithm of the target species concentration (100, 10, 1, and 0.1%). (**A**–**C**); each *C. longa* rhizome powders were mixed with three different plant powders (*Z. mays*, *O. sativa*, and *T. aestivum*) by ten-fold dilutions (0.1, 1, 10 and 100%, final mass of 2g) and the each mixture gDNA(10 ng/uL) was amplified using the *C. longa* targeting primer sets (CL_matK, CL_atpF, and CL_ycf2). The green dotted line means the 0.1% binary mixture Cts amplified using the *C. longa* targeting primer sets, CL_matK, CL_atpF and CL_ycf2) (**A**) binary mixture of *C. longa* and Z. mays; (**B**) binary mixture of *C. longa* and *O. sativa*; (**C**) binary mixture of *C. longa* and *T. aestivum*. (**D**) *Z. mays* powders were mixed with *C. longa* rhizome powders by ten-fold dilutions (0.1, 1, 10 and 100%, final mass of 2g) and each mixture gDNA(10 ng/uL) was amplified using the *Z. mays* targeting primer sets (ZM_matK, ZM_atpF, and ZM_ycf2). The blue dotted line means the 0.1% binary mixture Cts amplified using the *Z. mays* targeting primer sets, ZM_matK, ZM_atpF and ZM_ycf2). The real-time PCRs were carried out in triplicate (*n* = 3).

Globally, most *C. longa*-containing foods are prepared with rhizomes and dry powders. Therefore, we tested the sensitivity and specificity of the designed *C. longa* primer sets with binary mixtures (0.1–100% (*w*/*w*)) of *C. longa* dry rhizome powders containing each of three starch crops, including corn, rice, and wheat (Figure 3A–C). All three *C. longa* primer sets with slopes ranging from −3.35 to −3.550 exhibited *R*^2^ > 0.99 and efficiency values of 91.29–98.84% when used on mixed powders of *C. longa* and each starch crop, supporting the high sensitivity of the primer sets for verifying the presence of *C. longa* in mixtures sets (Supplementary Table S3). Subsequently, sensitivity of the three *Z. mays* primer sets was tested with binary mixtures of *Z. mays* and *C. longa* (0.1–100% (*w*/*w*)). Similarly, the three *Z. mays* primer sets with slope ranging from −3.12 to −3.44 exhibited *R*^2^ > 0.99 and efficiency values of 95.30–109.18% when used on mixed powders of *C. longa* and *Z. mays*, supporting the high sensitivity of the primer sets for verifying the presence of *Z*. *mays* in mixtures. Next, we determined the cut-off of Ct values based on the binary mixture standard collinearity equation of each primer set (Supplementary Table S3) to identify intended additions of cheap starch ingredients, such as *Z. mays*, in the *C. longa* powders. Ct values of 0.1% target species were determined as cut-off values for each primer set because additions of less than 0.1% of non-target species were not considered to be intended for illegal economic profit. The cut-off Ct values (0.1% target species in binary mixtures) were established to verify the presence of the target species from the calibration curves (Figure 3). The cut-off Ct values ranged from 26.82 to 29.59 cycles for each primer set targeting *C. longa* and 27.58 to 29.68 cycles for those targeting *Z. mays* (Supplementary Table S3).

Subsequently, we conducted a specificity test using the species-specific primer sets. A total of 10 species of cereals and vegetables were examined to assess cross-reactivity (Table 2). The cheap starch crops such as barley, wheat, oats, rice, sweat potato, and cassava, which are likely to be intentionally mixed as ingredients in complex turmeric foods for illegal economic profits, were included for the specificity test. In addition, one vegetable crop such as cabbage and one oilseed crop such as peanuts were used for the specificity test as out groups. 18S plant rRNA primer sets were used as a positive control [32], which exhibited lower Ct values than the cut-off. As shown in Table 2, Cl_matK, CL_atpF, and CL_ycf2 exhibited *C. longa*-specific amplification but did not amplify the DNA of other species. Similarly, ZM_matK, ZM_atpF, and ZM_ycf2 exhibited *Z. mays* specific amplification did not amplify the DNA of other species. The specificity test demonstrates that the primer sets could be useful for detecting the target species in unknown-ingredient powders and in complex food products.

### 3.4. Application of the Developed Real-Time PCR Assay to Blind Samples 

A blind test was conducted to estimate the reliability of the developed real-time PCR assays. Twenty unknown powder samples of *C. longa* and *Z. mays* were mixed randomly by an independent research group. The 18S rRNA plant primer sets were used as positive amplification controls [32], which exhibited low Cts (13.27–16.21; Table 3).

Next, we determined whether *Z. mays* powder was present in the samples based on the cut-off Ct values of the designed primer sets (0.1% *Z. mays* in binary mixtures). As a result, we identified four samples (sample 3, 9, 12, and 19) with Ct values exceeding the cut-off Ct values, indicating that the samples did not contain *Z. mays* powder mixed in *C. longa* powder. The other 16 samples were exhibited lower Cts than the cut-off Ct values, indicating that those samples contained *Z. mays* powder. In addition, the ratio of *Z. mays* powder mixed into the 16 samples was predicted using the developed binary mixture assay of the three primer sets (ZM_matK, ZM_atpF, and ZM_ycf2). The predicted percentage of *Z. mays* in each blind sample was extrapolated by inserting the Cts into the standard collinearity equation of each primer set (ZM_matK, ZM_atpF, and ZM_ycf2). As a result, the predicted percentages of *Z. mays* present in each sample were consistent with those of the mixed samples (Table 3). Therefore, the real-time PCR methodologies developed in this study demonstrated high accuracy for detecting the addition of *Z. mays* in *C. longa* rhizome powders. 

### 3.5. Application of the Developed Assay in Commercial Products

To verify adulteration with corn powder of *C. longa* food products, we performed the developed real-time PCR assays on 10 *C. longa* commercial food products (Supplementary Table S4, Table 4). First, the quality of genomic DNA isolated from the food products was evaluated using a spectrometer. As depicted in Table 4, the 18S rRNA primer sets exhibited low Cts (14.01–19.82), indicating that the gDNA from all the commercial products was sufficient to provide amplifiable gDNA. We found that all *C. longa* commercial food products (samples 1–10) were amplified with lower Ct values (from 14.1 to 21.971 cycles) using the *C. longa* species-specific primers (CL_matK, CL_atpF, and CL_ycf2) than the cut-off Ct values (Ct values of 0.1% *C. longa-*specific primer set in binary mixtures) for each primer set (CT values of CL_matK, CL_atpF, and CL_ycf2 were 28.65, 28.60, and 29.59 cycles, respectively; Figure 3, Supplementary Table S3). Additionally, all samples were amplified with higher Ct values (from 30.23 cycles to not detected before 40 cycles) with *Z. mays* targeting primers (ZM_matK, ZM_atpF, and ZM_ycf2) than the cut-off Ct values (Ct values of 0.1% *Z. mays-*specific primer sets in binary mixtures) for each primer set (28.41, 29.68, and 27.58 cycles, respectively; Supplementary Table S2). As a result, the commercial products purchased from local markets did not contain *Z. mays*, suggesting that the developed real-time PCR assays could be successfully applied to detect the presence of *Z. mays* in commercial complex *C. longa* products.

## 4. Conclusions

A real-time PCR assay is a highly sensitive, rapid, and specific method to detect target-species in processed food complexes. We designed three chloroplast gene targeted primer sets for both *C. longa* and *Z. mays*. To assess the quantities of the target-species present, standard curves were constructed using recombinant plasmid DNA and binary DNA mixtures. The specificities of the designed primers were confirmed with ten other species. Blind sample analysis and the application to commercial *C. longa* food products supported the effectiveness of the real-time PCR assays to detect *Z. mays* products added for illegal economic profits. Therefore, the developed real-time PCR assay could contribute to food safety and the protection of consumer’s rights. 

## Figures and Tables

**Table 1:** Primer sets designed for species-specific targeting.

Target Species	Target Gene	Primer	Length (bp)	Sequence (5′→3′)	Size (bp)	Tm (°C)
All plants	18s rRNA region	18s rRNA_F	25	TCTGCCCTATCAACTTTCGATGGTA	137	58
		18s rRNA_R	25	AATTTGCGCGCCTGCTGCCTTCCTT		
*Curcuma longa*	*matK*	*CL_matK_*F	19	CAATCCTATATGGTTGAGA	171	55
		*CL_matK_*R	18	GTCAGAAGACTCTATGGA		
	*atpF*	*CL_atpF_*F	20	GCATTATTGGTTGATAGAGA	194	58
		*CL_atpF_*R	22	GTTTATTTCAAGAATAGGATGG		
	*ycf2*	*CL_ycf2_*F	20	GAAGAAGAGGAAGAGGACAT	80	60
		*CL_ycf2_*R	20	CATATTCTAGGAGCCCAAAC		
*Zea mays*	*matK*	*ZM_matK_*F	19	TTGATATCGAACATAATGC	135	55
		*ZM_matK_*R	16	ACATCTTCTGGAACCT		
	*atpF*	*ZM_atpF_*F	19	TGGAAGCAGATGAGTATCG	160	60
		*ZM_atpF_*R	18	TGTTGTCGGACCTGATTC		
	*ycf2*	*ZM_ycf2_*F	20	AAGAGGATGAGTTGTCAGAG	99	59
		*ZM_ycf2_*R	18	GCAAGAAGTCCGAATCAG		

**Table 2:** Results of the specificity test with other plants.

NO	Species	Plant Systems	*Curcuma longa*				*Zea mays*		
		*18s rRNA*	*CL_matK*	*Cl_atpF*	*CL_ycf2*	*ZM_matK*		*ZM_atpF*	*ZM_ycf2*
			28 ^a^ (Cycles)	28 (Cycles)	29 (Cycles)	28 (Cycles)		29 (Cycles)	28 (Cycles)
1	*Curcuma longa* (Turmeric)	*+*	+ ^b^	*+*	*+*	-		-	-
2	*Hodeum vulgare* (Barley)	*+*	- ^c^	-	-	-		-	-
3	*Avena sativa* (Oats)	*+*	-	-	-	-		-	-
4	*Triticum aestivum* (Wheat)	*+*	-	-	-	-		-	-
5	*Zea mays* (Corn)	*+*	-	-	-	*+*		*+*	*+*
6	*Oryza sativa* (Rice)	*+*	-	-	-	-		-	-
7	*Brassica oleracea* var. *capitate* (Cabbage)	*+*	-	-	-	-		-	-
8	*Ipomoea batatas* (Sweat potato)	*+*	-	-	-	-		-	-
9	*Arachis hypogaea* (Peanuts)	*+*	-	-	-	-		-	-
10	*Manihot esculenta* (Cassava)	*+*	-	-	-	-		-	-

^a^ Cycles, conventional PCR cycles based on cut-off (Ct) values of each specific primer sets (Ct values of 0.1% binary mixture ); ^b^ +, detected at less than Ct values of primers; ^c^ -, not detected before the primers′ Ct values.

**Table 3:** Results of the blind mixture test for evaluating the reliability of the developed primer sets.

No.	Ingredient		PAC^a^	E^b^ (%)	*Z. mays* Specific Primer Ct ± SD			A/D
	*C. longa (%)*	*Z. mays (%)*			ZM_matK	ZM_atpF	ZM_ycf2	
1	99	1	13.57 ± 0.04	0.5–1.5	25.02 ± 0.06	25.61 ± 0.20	26.44 ± 0.21	A^d^
2	98	2	14.73 ± 0.09	1–5	25.09 ± 0.02	24.78 ± 0.12	22.8 ± 0.18	A
3	100	0	14.03 ± 0.01	ND^c^	31.51 ± 0.15	31.32 ± 0.26	35.15 ± 0.05	A
4	98	2	14.31 ± 0.01	1–5	24.40 ± 0.01	24.75 ± 0.15	23.92 ± 0.09	A
5	95	5	14.05 ± 0.01	1–5	23.72 ± 0.09	24.34 ± 0.09	21.94 ± 0.22	A
6	97	3	14.11 ± 0.06	1–5	23.45 ± 0.05	24.90 ± 0.07	22.97 ± 0.25	A
7	99.5	0.5	14.21 ± 0.02	0.5–1.5	25.09 ± 0.05	26.95 ± 0.07	26.34 ± 0.15	A
8	98.5	1.5	14.12 ± 0.05	0.5–1.5	25.59 ± 0.03	26.32 ± 0.10	24.69 ± 0.19	A
9	100	0	14.33 ± 0.08	ND	31.67 ± 0.20	31.01 ± 0.80	34.89 ± 0.10	A
10	98.5	1.5	14.23 ± 0.03	0.3–2	25.13 ± 0.09	25.69 ± 0.10	24.21 ± 0.02	A
11	99.5	0.5	14.27 ± 0.01	0.1–1	26.06 ± 0.09	27.08 ± 0.10	26.19 ± 0.09	A
12	100	0	13.61 ± 0.12	ND	31.33 ± 0.28	30.98 ± 0.11	34.46 ± 0.26	A
13	97	3	14.31 ± 0.10	1–5	24.97 ± 0.09	24.37 ± 0.07	23.86 ± 0.13	A
14	98	2	13.22 ± 0.07	1–5	24.19 ± 0.09	26.14 ± 0.12	23.22 ± 0.09	A
15	96	4	13.33 ± 0.06	1–5	23.92 ± 0.15	24.26 ± 0.03	22.72 ± 0.27	A
16	99	1	14.26 ± 0.05	0.1–1	25.75 ± 0.10	26.75 ± 0.14	25.11 ± 0.10	A
17	93	7	13.95 ± 0.07	5–10	22.11 ± 0.11	23.99 ± 0.13	21.72 ± 0.11	A
18	90	10	13.74 ± 0.02	10–15	22.11 ± 0.10	21.99 ± 0.18	21.23 ± 0.13	A
19	100	0	16.21 ± 0.07	ND	30.82 ± 0.20	31.09 ± 0.01	34.06 ± 0.56	A
20	97	3	13.27 ± 0.1	1–5	23.96 ± 0.05	24.28 ± 0.10	23.22 ± 0.12	A

^a^ Positive amplification control (18s rRNA); ^b,^ expected ratio of the *Z. mays*; ^C,^ not detected; ^d^ accordance.

**Table 4:** Result of the real-time PCR assay using 10 commercial products.

Real Commercial Products Tested that Labeled as 100% *Curcuma longa*							
Sample Number	Plant System (18s rRNA)	*CL_matK*	*CL_atpF*	*CL_ycf2*	*ZM_matK*	*ZM_atpF*	*ZM_ycf2*
1	14.49	14.83	14.10	14.24	ND^a^	34.00	ND
	±0.15	±0.13	±0.17	±0.14		±0.07	
2	15.23	15.27	15.70	14.50	38.26	33.59	ND
	±0.06	±0.18	±0.09	±0.08	±1.1	±2.01	
3	19.82	22.62	22.65	20.16	36.28	32.32	34.79
	±0.08	±0.27	±0.04	±0.06	±0.61	±0.54	±0.47
4	15.25	15.95	16.33	15.25	33.60	30.05	34.35
	±0.06	±0.07	±0.03	±0.14	±0.20	±0.07	±0.21
5	14.01	15.29	15.46	15.29	31.53	31.41	ND
	±0.05	±0.07	±0.01	±0.07	±0.54	±0.56	
6	19.19	17.68	21.97	15.28	32.34	31.14	ND
	±0.03	±0.12	±0.04	±0.04	±0.76	±0.14	
7	16.44	16.10	16.39	14.90	34.89	34.57	ND
	±0.18	±0.03	±0.03	±0.09	±0.35	±0.65	
8	18.87	15.35	20.24	14.10	32.82	34.55	ND
	±0.08	±0.02	±0.12	±0.02	±0.33	±0.95	
*9*	15.31	14.63	15.15	13.26	31.05	34.00	ND
	±0.09	±0.08	±0.06	±0.02	±4.55	±0.08	
10	16.10	15.84	16.57	14.95	32.02	30.23	33.77
	±0.01	±0.03	±0.02	±0.06	±0.24	±0.06	±0.92

^a^ ND indicates not detected at less than 40 cycles.

## Supplementary Materials

The following are available online at https://www.mdpi.com/2304-8158/9/7/882/s1, Figure S1: Alignment of the target chloroplast gene (*matK*, *atpF* and *ycf2*) nucleotide sequences of *C. longa*, *Z. mays* and starch crops (*O. sativa* and *T. aestivum*) mainly eating as powders amplified by *C. longa* specific primer sets (CL_matK CL_atpF and CL_ycf 2), Figure S2: Alignment of the target chloroplast gene(*matK*, *atpF* and *ycf2*)nucleotide sequences of *Z. mays*, *C. longa* and starch crops(*O. sativa*, and *T. aestivum*) mainly eating as powders, amplified by *Z. mays* s pecific primer sets (ZM_matK, ZM_atpF and ZM_ycf2), Figure S3: Real time PCR with SYBR Green and DNA melting curve analyses.(A) Serial dilution series recombinant plasmids (10^7^–10^3^) containing *C. longa* specific gene (*matK*, *atpF* and *ycf 2*) sequence were amplified using *C. longa* specific primer sets. (**B**) Serial dilution series recombinant plasmids (10^7^–10^3^) containing *Z. mays* specific gene (*matK*, *atpF* and *ycf 2*) sequence were amplified using *Z. mays* specific primer sets. The real time PCRs were performed on a QuantStudio 3 Real Time PCR System (Applied Biosystems, Foster City, CA, USA) and carried out in triplicate (*n* = 3), Figure S4: Real time PCR with SYBR Green and DNA melting curve analyses green lanes mean the *C. longa* blue lanes mean *Z. mays* and pink lanes mean NTC. (**A**) Serial dilution series of C. *longa* genomic DNA (10 ng–1 pg) was amplified using *C*. *longa* specific primer sets. (**B**) Serial dilution series of *Z*. *mays* (10 ng–1 pg) was amplified using *Z. mays* specific primer sets. The real time PCRs were performed on a QuantStudio 3 Real Time PCR System (Applied Biosystems, Foster City, CA, USA) and carried out in triplicate (*n* = 3), Table S1: Evaluation of slope, *R*^2^, and efficiency using the developed primer sets, Table S2: Result of the real-time PCR assay in an interlaboratory experiment, Table S3: Evaluation of the slope, *R*^2^, and efficiency using binary mixtures containing three different intentionally added powders, Table S4: Information on the commercial food products.
