# Supplementary material for: Development and Validation of a Real-Time PCR Based Assay to Detect Adulteration with Corn in Commercial Turmeric Powder Products
Source: Foods. 2020 Jul 5;9(7):882. doi: 10.3390/foods9070882 (PMC7404567; doi:10.3390/foods9070882)
Supplement: Supplementary file 1 [file foods-09-00882-s001.zip › foods-845976-supplementary-tables.pdf]

Supplementary table 1. Evaluation of slope, R^2, and efficiency using the developed primer sets.

Target species	Primer	Recombinant plasmid			Genomic DNA		
		Y (Slope)	R^2	Efficiency (%)	Y (Slope)	R^2	Efficiency (%)
Curcuma longa	*CL_matK*	-3.50	1.00	93.14	-3.47	1.00	94.22
	CL_atpF	-3.14	1.00	108.40	-3.42	0.99	95.92
	CL_ycf2	-3.35	1.00	98.94	-3.47	1.00	94.09
Zea mays	*ZM_matK*	-3.48	1.00	93.72	-3.54	1.00	91.78
	ZM_atpF	-3.21	1.00	104.89	-3.45	1.00	94.96
	ZM_ycf2	-3.43	1.00	95.57	-3.53	1.00	92.16

Supplementary table 2. Result of the real-time PCR assay in an interlaboratory experiment.

Target species	Primer	Laboratory 1			Laboratory 2		
		Y (Slope)	R^2	Efficiency (%)	Y (Slope)	R^2	Efficiency (%)
Curcuma longa	*CL_matK*	-3.50	1.00	93.14	-3.47	1.00	94.22
	CL_atpF	-3.14	1.00	108.40	-3.42	0.99	95.92
	CL_ycf2	-3.35	0.99	98.94	-3.47	1.00	94.09
Zea mays	*ZM_matK*	-3.48	1.00	93.72	-3.54	1.00	91.78
	ZM_atpF	-3.41	1.00	96.32	-3.45	1.00	94.96
	ZM_ycf2	-3.43	1.00	95.57	-3.53	1.00	92.16

Supplementary table 3. Evaluation of the slope, R^2, and efficiency using binary mixtures containing three different intentionally added powders.

**Target species**	**Primer**	**Binary mixture with *Zea mays***				**Binary mixture with *Oryza sativa***				**Binary mixture with *Triticum aestivum***			
		**Y (Slope)**	**R^2**	**Efficiency (%)**	**Cut-off Cts**	**Slope**	**R^2**	**Efficiency (%)**	**Cut-off Cts**	**Slope**	**R^2**	**Efficiency (%)**	**Cut-off Cts**
Curcuma longa	CL_matK	-3.55	0.99	91.29	28.65	-3.5	0.99	93.07	27.08	-3.47	1	94.17	26.90
	CL_atpF	-3.54	1	91.64	28.60	-3.35	1	98.84	26.85	-3.53	1	91.99	26.82
	CL_ycf2	-3.44	1	95.3	29.59	-3.5	0.99	93.07	29.19	-3.38	1	97.63	28.87

Target species	Primer	Binary mixture with *Curcuma longa*			
		Y (Slope)	R^2	Efficiency (%)	Cut-off Cts
Zea mays	ZM_matK	-3.38	0.99	97.63	28.41
	ZM_atpF	-3.12	1	109.18	29.68
	ZM_ycf2	-3.44	1	95.3	27.58

Supplementary table 4. Information on the commercial food products.

Species	Sample	Sample type	Ingredients
Curcuma longa	1	Turmeric powder	*Curcuma longa* 100%
	2	Turmeric powder	*Curcuma longa* 100%
	3	Turmeric powder	*Curcuma longa* 100%
	4	Turmeric powder	*Curcuma longa* 100%
	5	Turmeric powder	*Curcuma longa* 100%
	6	Pill	*Curcuma longa* 49% Dried orange peel 41% Sticky rice 10%
	7	Turmeric powder	*Curcuma longa* 100%
	8	Turmeric powder	*Curcuma longa* 100%
	9	Turmeric powder	*Curcuma longa* 100%
	10	Turmeric powder	*Curcuma longa* 100%
