# Supplementary material for: Development and Validation of a Real-Time PCR Based Assay to Detect Adulteration with Corn in Commercial Turmeric Powder Products
Source: Foods. 2020 Jul 5;9(7):882. doi: 10.3390/foods9070882 (PMC7404567; doi:10.3390/foods9070882)
Supplement: Supplementary file 1 [file foods-09-00882-s001.zip › foods-845976-supplementary-figures.pdf]

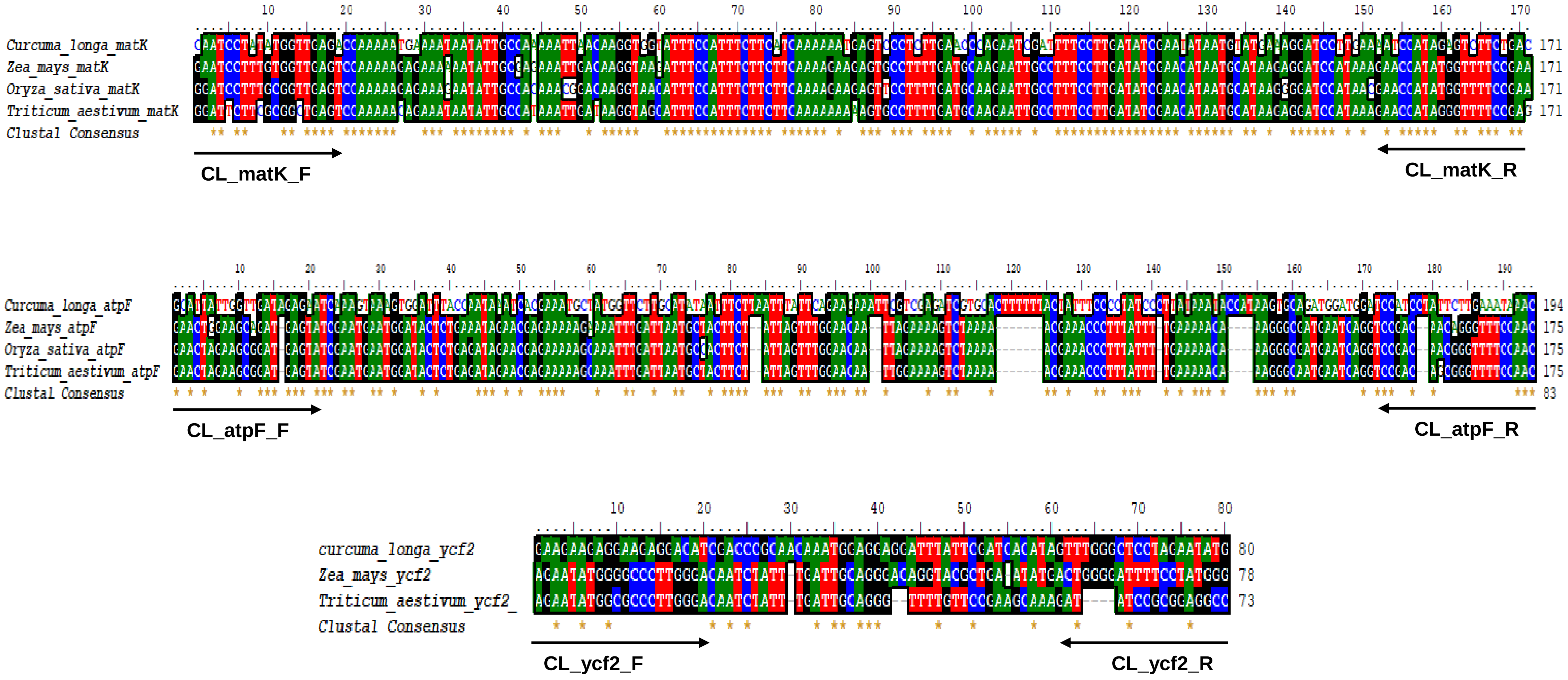

Supplementary Fig. 1 : Alignment of the target chloroplast gene (*matK*, *atpF* and *ycf2*) nucleotide sequences of *C. longa*, *Z. mays* and starch crops (*O. sativa*, and *T. aestivum*) mainly eating as powders amplified by *C. longa* specific primer sets (CL_matK, CL_atpF and CL_ycf2).

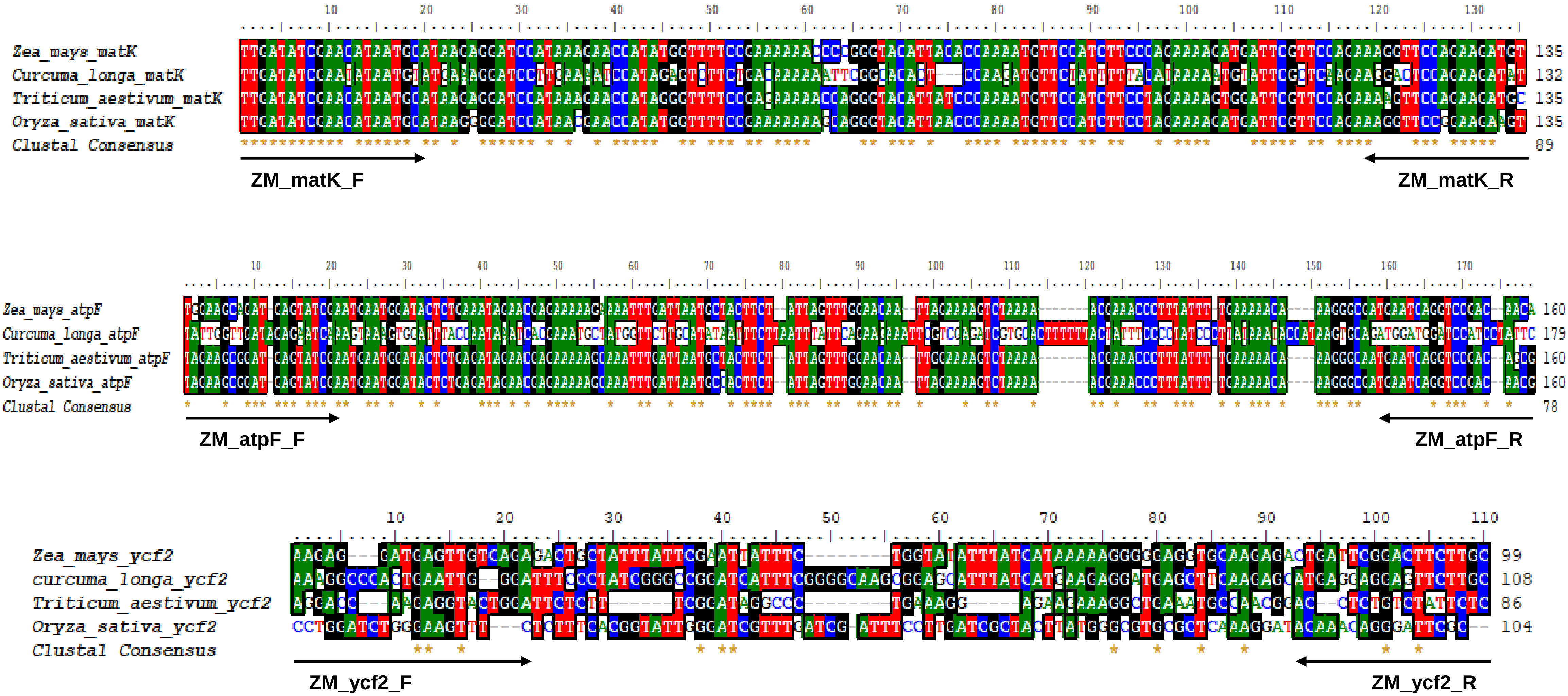

Supplementary Fig. 2 Alignment of the target chloroplast gene (*matK*, *atpF* and *ycf2*) nucleotide sequences of *Z. mays*, *C. longa* and starch crops (*O. sativa*, and *T. aestivum*) mainly eating as powders, amplified by *Z. mays* specific primer sets (ZM_matK, ZM_atpF and ZM_ycf2).

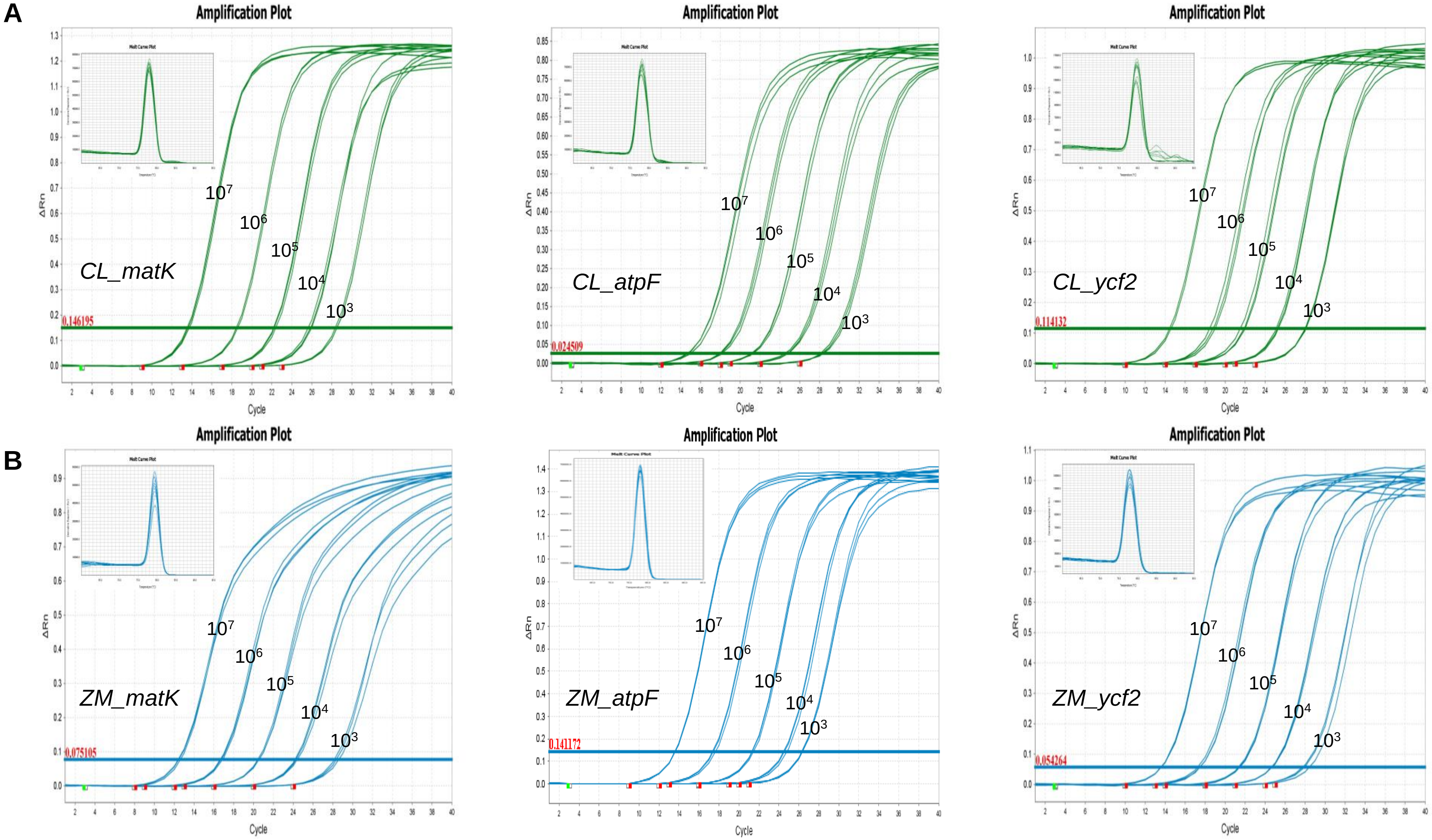

Supplementary Fig. 3 Real-time PCR with SYBR Green and DNA melting curve analyses. (A) Serial dilution series recombinant plasmids (10^7 – 10^3) containing *C. longa* specific gene (*matK*, *atpF* and *ycf2*) sequence were amplified using *C. longa* specific primer sets. (B) Serial dilution series recombinant plasmids (10^7 – 10^3) containing *Z. mays* specific gene (*matK*, *atpF* and *ycf2*) sequence were amplified using *Z. mays* specific primer sets. The real-time PCRs were performed on a QuantStudio 3 Real-Time PCR System (Applied Biosystems, Foster City, CA, USA) and carried out in triplicate (n=3).

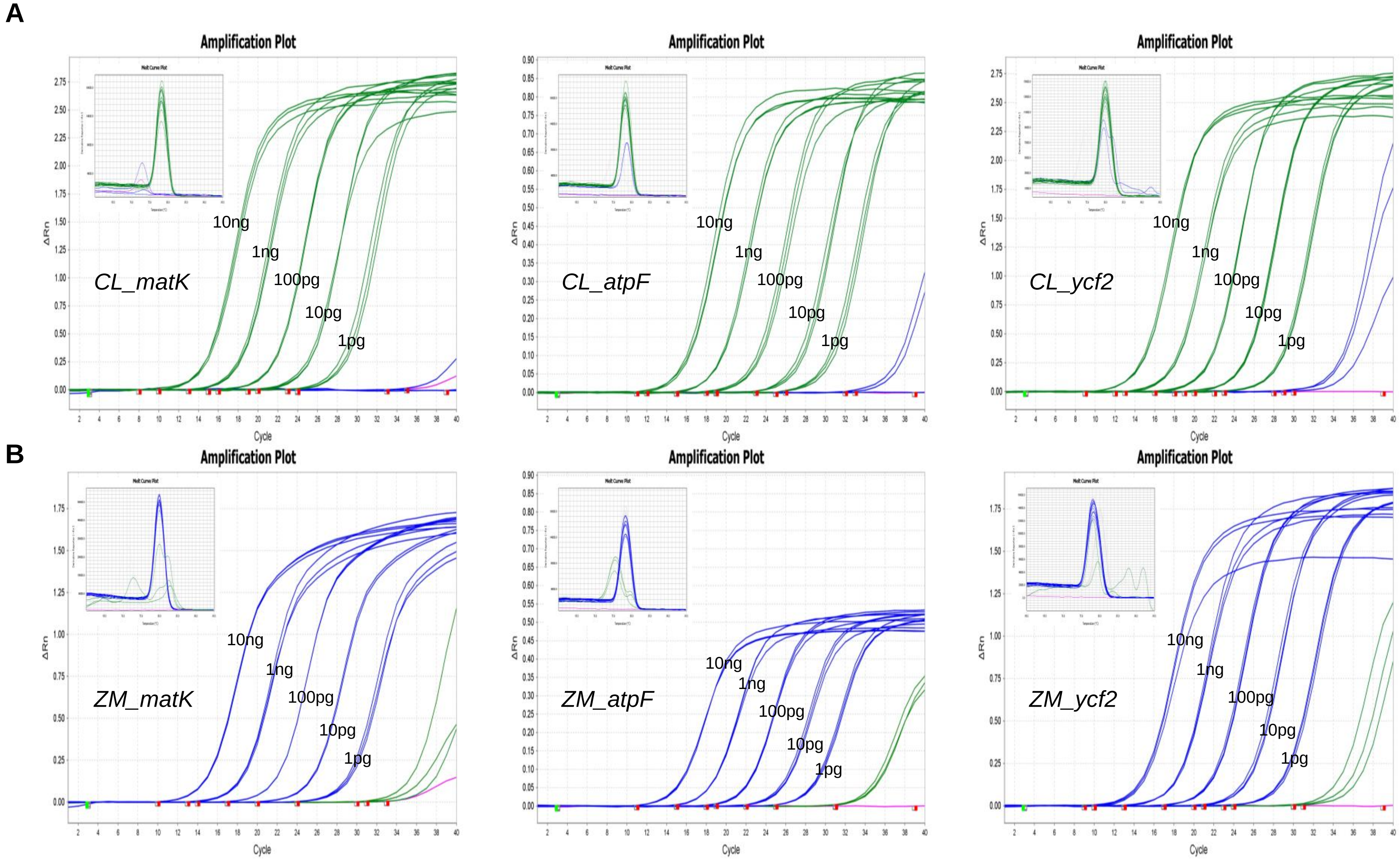

Supplementary Fig. 4 Real-time PCR with SYBR Green and DNA melting curve analyses; green lanes mean the *C. longa* blue lanes mean *Z. mays* and pink lanes mean NTC. (A) Serial dilution series of *C. longa* genomic DNA (10ng – 1pg) was amplified using *C. longa* specific primer sets. (B) Serial dilution series of *Z. mays* (10ng – 1pg) was amplified using *Z. mays* specific primer sets. The real-time PCRs were performed on a QuantStudio 3 Real-Time PCR System (Applied Biosystems, Foster City, CA, USA) and carried out in triplicate (n=3).
